# *Ginkgo biloba* extract EGb 761^®^ improves cognition and overall condition after ischemic stroke: Results from a pilot randomized trial

**DOI:** 10.3389/fphar.2023.1147860

**Published:** 2023-03-29

**Authors:** Mei Cui, Tongyao You, Yuwu Zhao, Ruozhuo Liu, Yangtai Guan, Jianren Liu, Xueyuan Liu, Xin Wang, Qiang Dong

**Affiliations:** ^1^ Department of Neurology, Huashan Hospital, Fudan University, Shanghai, China; ^2^ Department of Neurology, Shanghai Jiao Tong University Affiliated Sixth People’s Hospital, Shanghai, China; ^3^ Department of Neurology, Chinese PLA General Hospital, Beijing, China; ^4^ Department of Neurology, Changhai Hospital, Naval Medical University, Shanghai, China; ^5^ Department of Neurology, Shanghai Jiao Tong University Affiliated Ninth People’s Hospital, Shanghai, China; ^6^ Department of Neurology, Shanghai Tong Ji University Affiliated Tenth People’s Hospital, Shanghai, China; ^7^ Department of Neurology, Zhongshan Hospital, Fudan University, Shanghai, China

**Keywords:** Ginkgo biloba extract, ischemic stroke, cognitive function, post-stroke recovery, randomized trial

## Abstract

**Background:** Patients who experienced an ischemic stroke are at risk for cognitive impairment. Quantified *Ginkgo biloba* extract EGb 761^®^ has been used to treat cognitive dysfunction, functional impairment and neuropsychiatric symptoms in mild cognitive impairment and dementia.

**Objectives:** To assess the cognitive-related effects of EGb 761^®^ treatment in patients after acute ischemic stroke, as well as the feasibility of patient selection and outcome measures.

**Methods:** We conducted a randomized, multicentric, open-label trial at 7 centers in China. Patients scoring 20 or lower on the National Institutes of Health Stroke Scale were enrolled between 7 and 14 days after stroke onset and randomly assigned to receive 240 mg per day of EGb 761^®^ or no additional therapy for 24 weeks in a 1:1 ratio. Both groups received standard treatments for the prevention of recurrent stroke during the trial. General cognitive function and a battery of cognitive tests for sub-domains were evaluated at 24 weeks. All patients were monitored for adverse events.

**Results:** 201 patients ≥50 years old were included, with 100 assigned to the EGb 761^®^ group and 101 to the reference group. The mean change from baseline on the global cognitive function as assessed by the Montreal Cognitive Assessment score was 2.92 in the EGb 761^®^ group and 1.33 in the reference group (between-group difference: 1.59 points; 95% confidence interval [CI], 0.51 to 2.67; *p* < 0.005). For cognitive domains, EGb 761^®^ showed greater effects on the Hopkins Verbal Learning Test Total Recall (EGb 761^®^ change 1.40 vs. reference −0.49) and Form 1 of the Shape Trail Test (EGb 761^®^ change −38.2 vs. reference −15.6). Potentially EGb 761^®^-related adverse events occurred in no more than 3% of patients.

**Conclusion:** Over the 24-week period, EGb 761^®^ treatment improved overall cognitive performance among patients with mild to moderate ischemic stroke. Our findings provide valuable recommendations for the design of future trials, including the criteria for patient selection.

**Clinical Trial Registration:** www.isrctn.com, identifier ISRCTN11815543.

## 1 Introduction

Patients surviving stroke are experiencing cognitive impairment faster than those of stroke-free controls and several large population-based studies have reported significant cognitive decline in long-term follow-up (Wang et al., 2012; Levine et al., 2015; Zheng et al., 2019; Lo et al., 2022). To date, the mechanisms, magnitude and predictors of cognitive decline after stroke is still incompletely understood. Clinical characteristics are a key determinant of the variability in dementia incidence among post-stroke patients. The presence of lesion burden, multiple acute infarcts and total infarct volume, all have been shown to predict post-stroke cognitive impairment independently from demographic and vascular risk factors (Pendlebury and Rothwell, 2009; Pendlebury and Rothwell, 2019). There is also a stepwise association between the manifestation of severe small vessel diseases (SVD) or multiple lacunes and delayed-onset dementia after stroke (Mok et al., 2016). Moreover, vascular factors contribute to dementia through cerebral infarcts and white matter changes (Pendlebury and Rothwell, 2019). In particular, Foster *et al.* have provided clinicopathological evidence that pyramidal neuron atrophy in the dorsolateral prefrontal cortex, rather than loss of neuronal numbers, was associated with distinct cognitive deterioration in post-stroke dementia and vascular dementia (Foster et al., 2014). Similarly, neuropsychiatric symptoms (NPS) such as depression, irritability, agitation, apathy and anxiety have also been found in significant proportions (23%–33%) of stroke patients (Angelelli et al., 2004; Zhang et al., 2013; Karakus et al., 2017; Salinas et al., 2017; Villa et al., 2018). Acute stroke treatment is typically followed by secondary prevention in accordance with current guidelines (Wang et al., 2017). This mainly consists of antithrombotic treatments (anti-platelet drugs, anticoagulants), and management of risk factors (such as hypertension, hyperlipidemia, metabolic syndrome or diabetes mellitus). There are limited pharmacological therapies to address subsequent cognitive impairment aside from brain stimulation techniques and physical exercise (Bordet et al., 2017), and little attention has been paid to cholinesterase inhibitors and memantine. Only one randomized controlled trial (RCT) showed the effectiveness of Actovegin on cognitive outcomes in patients with mild-to-moderate ischemic stroke (Guekht et al., 2017), highlighting the need for more evidence from large prospective cohorts. Some patients with post-stroke depression responded to selective serotonin inhibitors, whereas treatments of other NPS appears to be less straightforward (FOCUS Trial Collaboration, 2019; Hankey, 2020; Lundström, 2020).

*Ginkgo biloba* extract EGb 761^®^ is a standardized product prepared to a ratio of 35–67:1 from dry extract from Ginkgo biloba leaves to final extract, extraction solvent: acetone 60% (weight/weight) (DeFeudis, 2003). It contains 22.0%–27.0% ginkgo flavonoids calculated as ginkgo flavone glycosides and 5.0%–7.0% terpene lactones consisting of 2.8%–3.4% ginkgolides A, B, C and 2.6%–3.2% bilobalide, 7% proanthocyanidins, certain low-molecular-weight organic acids, and less than 5 ppm ginkgolic acids (Müller et al., 2012). It is commercially available as tablets and drops and freshly dissolved in growth medium (EGb 761^®^ is a registered trade mark of Dr. Willmar Schwabe GmbH & Co. KG, Karlsruhe, Germany) (Koltermann et al., 2009). EGb 761^®^ has been proven to in clinical studies enhance perfusion and decrease blood viscosity (Müller et al., 2012). Randomized, placebo-controlled clinical trials have demonstrated that EGb 761^®^ improved cognitive performance and neuropsychiatric symptoms in patients with age-associated mild impairment in cognitive function, vascular dementia and Alzheimer’s disease (Gavrilova et al., 2014; Li et al., 2018). Meanwhile, an injectable form of the extract reduced the infarct size, neurological deficits, and further restored motor function with mitochondrial dynamics in a rat model of stroke (Li et al., 2018). These findings suggest that EGb 761^®^ may have a role in the prevention and treatment of cognitive impairment following stroke.

Since there is still a lack of effective drugs that can reliably prevent or treat cognitive decline in this population, we designed this pilot trial to assess whether EGb 761^®^ 240 mg/day for 24 weeks would confer cognitive benefits after an acute stroke; we also planned to evaluate the effect of EGb 761@ in various cognitive domains and on NPS, as well as obtain data which can inform the process of designing a randomized controlled trial with oral EGb 761^®^ treatment in post-stroke cognitive impairment.

## 2 Methods

### 2.1 Study design and setting

We conducted a parallel-group, randomized, multicentric, open-label pilot trial at 7 centers of China in accordance with international (International Conference on Harmonization, ICH) and national guidelines for Good Clinical Practice and applicable laws of the People’s Republic of China. Details around the centers are described in the supplementary material (Supplementary Table S1). It was approved by the ethics committees of all participating clinical sites. Two amendments to the protocol were also approved by all ethics committees. Informed consent was obtained from all patients before enrolment in the trial. Clinical trials registration: study ID ISRCTN11815543 on the ISRCTN registry.

### 2.2 Inclusion and exclusion criteria

Patients of both sexes, at least 50 years old, who had given informed consent and scored no higher than 20 on the National Institutes of Health Stroke Scale (NIHSS) were enrolled during 7–14 days after an acute stroke (Lyden, 2017). Their MRI scans had to indicate acute ischemic cerebral infarction and rule out signs of hemorrhage, tumor, normal pressure hydrocephalus or other serious cerebral disorder. Lacunes, white matter hyperintensities or mild atrophy, or combinations thereof consistent with pre-dementia Alzheimer’s disease or cerebrovascular disease were acceptable. Patients had to be able to understand and respond to interview questions, complete questionnaires and take part in neuropsychological testing with the necessary language skills. Each patient needed a regular contact person (e.g., partner, close relative, friend) who was willing to accompany him/her to provide information about the patient’s cognitive problems, functional abilities and neuropsychiatric symptoms during the hospital visits.

Patients were excluded from the study if they had any type of dementia or other major neurological disorder (e.g., Parkinson’s, Huntington’s, Pick’s or Creutzfeldt-Jakob disease, seizure disorder), psychiatric disorder (e.*g., major* depression, generalized anxiety disorder), alcohol or substance abuse/addiction, severe and uncontrolled cardiovascular disease, severe renal or hepatic dysfunction, insufficiently controlled diabetes mellitus, clinically significant thyroid dysfunction, vitamin B12 or folic acid deficiency, HIV or syphilitic infection, active malignant disease or any gastrointestinal disease with impaired absorption of orally applied drugs. Moreover, other criteria for exclusion were long-term hospitalization, aphasia, dysarthria, paresis of the dominant upper extremity, severe and insufficiently corrected loss of vision or hearing, severe language difficulties and any other disability that could have prevented valid cognitive testing. Female patients of childbearing potential and patients with known sensitivity to Ginkgo biloba extract were also excluded. In addition, patients who were taking psychoactive drugs (e.g., antidepressants, neuroleptics), anticholinergic drugs, anti-epileptics, anti-coagulants, anti-dementia drugs, cognition enhancers or perfusion-enhancing agents were not allowed to take part in the study.

### 2.3 Randomization and treatment

Randomization by means of a validated computer program (SAS macro RANSCH) was performed by a member of the biometrics department who was not otherwise involved in conducting of the trial. Eligible patients were identified by the clinician conducting the assessments. Randomization numbers were allocated to the patients in the order of inclusion. To minimize allocation bias, treatment information (EGb 761^®^ or reference group) was contained in sealed envelopes matched to the randomization numbers.

All patients received standard treatment in accordance with current guidelines for the prevention of stroke recurrence, including general supportive care, antiplatelet drugs and treatment for acute complications (Liu et al., 2020); nootropic agents were not allowed for use in this trial. Patients randomized to EGb 761^®^ treatment took 2 tablets at 40 mg EGb 761^®^ three times a day, for a daily dose of 240 mg in addition to standard treatment for a period of 24 weeks. Those randomized to the reference group received standard treatment only.

### 2.4 Outcome measures

Cognitive assessments: the main interest of this study was the change in cognitive status from baseline assessed by the validated Beijing version of the Montreal Cognitive Assessment (MoCA) at 24 weeks. The MoCA assesses a number of sub-domains of cognition (visuospatial/executive, naming, memory, attention, language, abstraction, delayed recall, orientation) (Julayanont and Nasreddine, 2017). Scores range from 0–30, with lower scores indicating more severe impairment. One point is added for patients who have less than 12 years of education. Cognitive impairment was defined as MoCA <26 (Nasreddine et al., 2005). The cognitive domains evaluated also included the changes in Hopkins Verbal Learning Test–Revised (HVLT-R), a scale that measures verbal learning and memory with lower scores indicating worse functioning (Shi et al., 2012); the Shape Trail Test (STT), checking the set shifting of executive function, with higher scores predicting worse ability (Zhao et al., 2013); a category version of the Verbal Fluency Test (VFT), which assesses the role of frontal lobe function by generating exemplars in the given category within 1 min (Mok et al., 2004); the Digit Symbol Substitution Test (DSST) of the Wechsler Adult Intelligence Scale—Revised (WAIS-R), a test for associative learning by correctly matching symbols to numbers within limited time (Jaeger, 2018).

Clinical status was assessed using the following instruments: the Neuropsychiatric Inventory (NPI), a multidimensional scale for the assessment of neuropsychiatric symptoms, the total composite score ranging from 0 to 144, where higher scores indicates greater abnormalities (Wang et al., 2012); the Hospital Anxiety and Depression scale (HADS), with higher scores indicating more psychological distress (Leung et al., 1999); the Clinical Global Impression of Change (CGI-C), targeting overall change in the patients’ mental health within the context of clinical experience (Busner and Targum, 2007); National Institutes of Health Stroke Scale (NIHSS), a measure of patients’ neurological status as well as stroke severity (Chalos et al., 2020); and incidence of recurrent stroke. Safety outcomes included vital signs, physical examination, 12-lead electrocardiograms, laboratory tests and recording of adverse events. All efficacy and safety outcomes were documented at baseline, at week 12 and 24. The members of an independent clinical-event adjudication committee confirmed all the assessments since they were unaware of the trial assignments.

### 2.5 Statistical analysis

Taking into account the exploratory nature of the trial, a sample size of 200 patients (100 per treatment group, 1:1 randomization, assumed drop-out rate of 10%) was chosen to achieve a statistical power of 80% to detect a minimum standardized difference of 0.5 within a two-group multivariate repeated measures design for a two-sided test, three time points, 3 variables at a descriptive significance of α = 0.05.

The outcome analyses were predefined using data from the full analysis set (FAS) population. For comparisons within and between treatment groups, two-sided *p*-values were calculated, applying the *t*-test to quantitative variables (paired *t*-test for within-group comparisons) and Fisher’s exact test to qualitative variables. Missing values were replaced by the last observation carried forward method. Statistical analysis was performed using SAS software, version 9.2 and higher (SAS Institute Inc., Cary, NC, United States) and SPSS version 24.0 (SPSS Inc., Chicago, IL, United States, 2001).

## 3 Results

### 3.1 Characteristics of the study population

**FIGURE 1:**
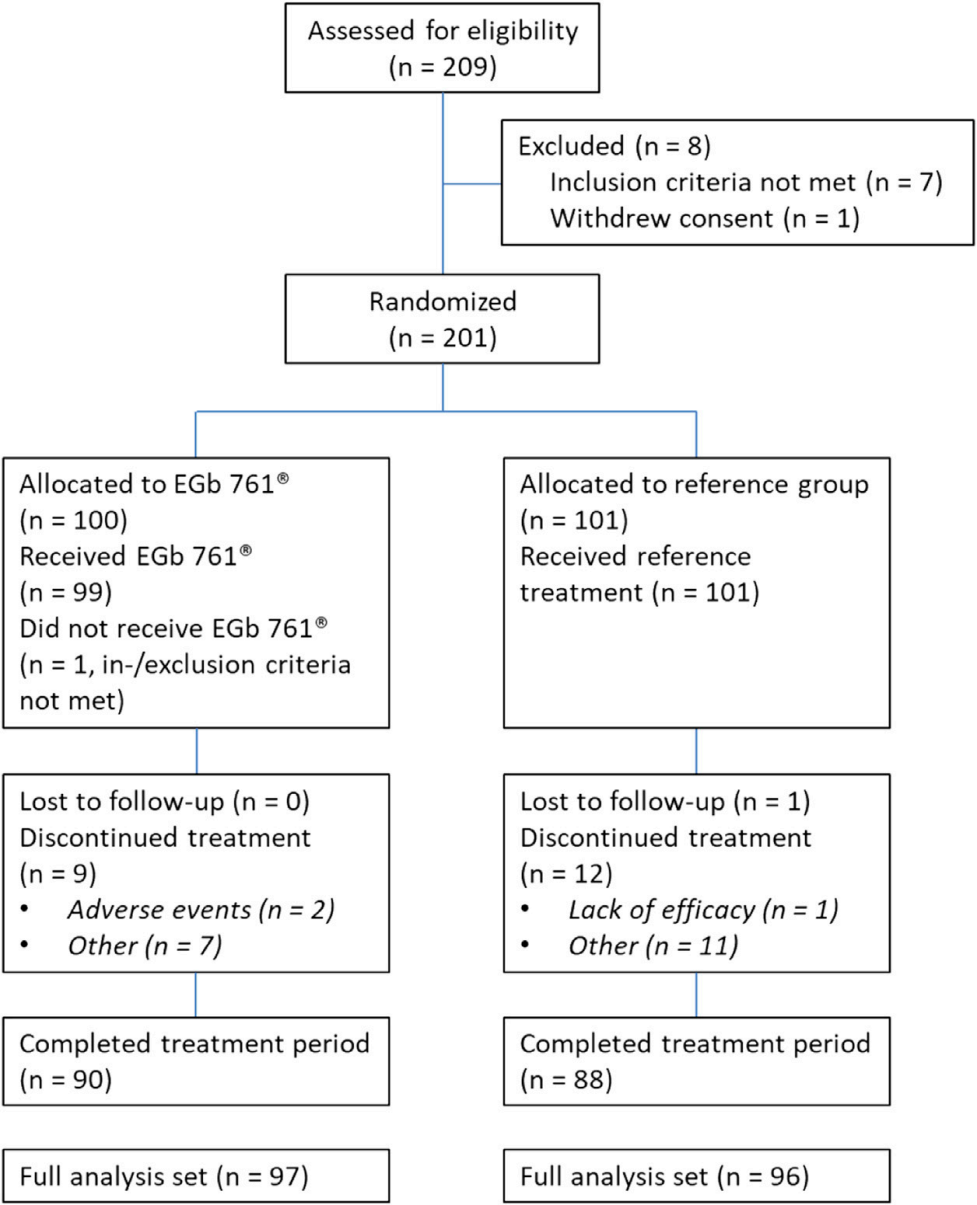
Patients flow diagram.

Between January 2014 and May 2016, 209 patients were screened for the study. Two hundred one were enrolled and underwent randomization along with standard treatment, with 100 to the EGb 761^®^ group and 101 to the reference group (no additional treatment). Figure 1 shows the patient flow chart. Overall, 23 patients discontinued the trial. The full analysis set (FAS) for the evaluation of treatment effects (EGb 761^®^ group, n = 97; reference group, n = 96) comprised all randomized patients who had at least one cognitive test result or one NPS rating after baseline and all patients randomized to receive EGb 761^®^ who terminated the trial early due to lack of efficacy or an adverse event for which a causal relationship with the study drug could not be ruled out. All patients of the reference group (n = 101) and all patients of the EGb 761^®^ group who took at least one dose of the study drug (n = 99) were included in the safety analysis (safety population, SAF).

Demographic characteristics and scales at baseline were similar between two groups except for the Neuropsychiatric Inventory (NPI) total score (Table 1). The numerically higher mean score and the high standard deviation in the reference group were due to a small number of outliers with high scores. The mild mean National Institutes of Health Stroke Scale (NIHSS) score indicated minimal neurological deficits and those slight differences between groups did not reach statistical significance.

**TABLE 1:** Characteristics of patients at baseline; n (%) or mean (SD); two-sided *p*-values of *t*-test (continuous variables) or Fisher’s exact test (categorical variables).

	EGb 761^®^ (n = 97)	Standard treatment (n = 96)	*p*-value
Female [n, %]	26 (26.8%)	20 (20.8%)	0.3988
Male [n, %]	71 (73.2%)	76 (79.2%)	
Age (years) [mean, SD]	62.6 ± 8.3	64.1 ± 8.3	0.2026
NIHSS total score [mean, SD]	1.98 ± 2.22	2.18 ± 2.23	0.5384
MoCA total score [mean, SD]	23.02 ± 4.68	23.47 ± 4.26	0.4876
HVLT total recall [mean, SD]	16.53 ± 4.53	17.20 ± 5.64	0.3628
STT trail 1 [mean, SD]	121.5 ± 109.5	109.7 ± 67.28	0.3669
STT trail 2 [mean, SD]	234.8 ± 176.6	239.6 ± 196.0	0.8588
VFT [mean, SD]	12.91 ± 5.00	12.44 ± 4.22	0.4814
WAIS-R DSST [mean, SD]	25.58 ± 11.97	25.58 ± 12.97	0.9973
NPI total score [mean, SD]	4,90 ± 13,04	8,88 ± 54,83	0.4906
HADS-Anxiety [mean, SD]	4.79 ± 3.99	5.27 ± 3.98	0.4066
HADS-Depression [mean, SD]	4.92 ± 4.01	5.08 ± 4.01	0.7742

Abbreviations: NIHSS, national institutes of health stroke scale; MoCA, montreal cognitive assessment; HVLT, hopkins verbal learning test; STT, shape trail test; VFT, verbal fluency test; WAIS-R, Wechsler Adult Intelligence Scale-Revised; DSST, digit symbol substitution test; NPI, neuropsychiatric inventory; HADS, hospital anxiety and depression scale; SD, standard deviation.

### 3.2 Efficacy

**FIGURE 2:**
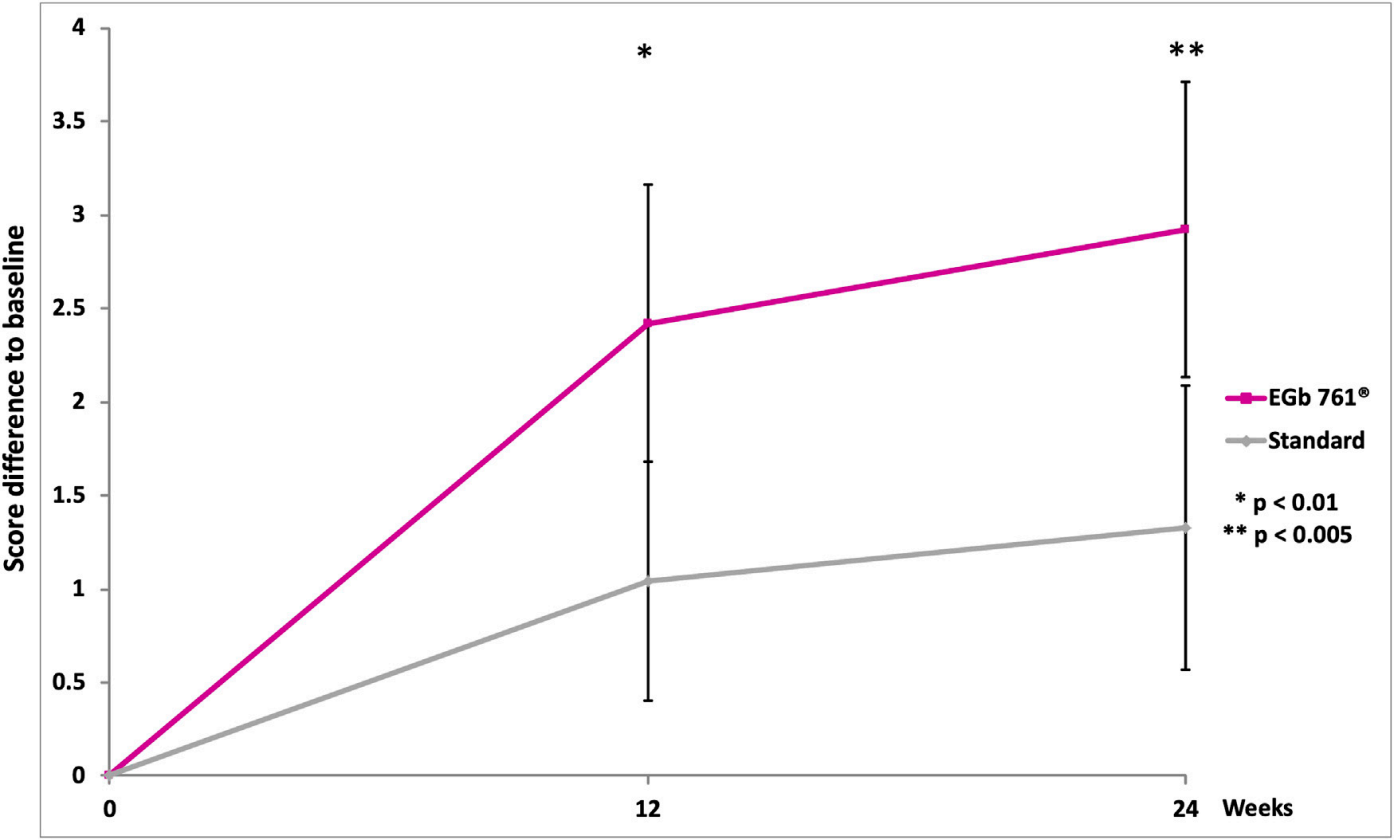
Change in Montreal Cognitive Assessment (MoCA) scores over the course of the study (means, 95% confidence intervals, two-sided *p*-values of *t*-test for between-group differences).

A significant difference in total MoCA scores favoring EGb 761^®^ was observed at 24 weeks. The mean change from the baseline MoCA rating at week 24 was 2.92 in the EGb 761^®^ group and 1.33 in the reference group (drug-reference control difference: 1.59 points; 95% confidence interval [CI], 0.51 to 2.67; *p* < 0.001; Table 2; Figure 2). For three sub-domains of the MoCA, scores of delayed recall, orientation and language in the EGb 761^®^ group at 24 weeks showed significantly larger improvements (delayed recall: EGb 761^®^ change 0.88 vs. reference 0.17, 95% CI, 0.31 to 1.10, *p* < 0.001; orientation: EGb 761^®^ change 0.28 vs. reference −0.11, 95% CI, 0.18 to 0.60, *p* < 0.001; language: EGb 761^®^ change 0.45 vs. reference 0.09, 95% CI, 0.06 to 0.66, *p* < 0.05).

**TABLE 2:** Efficacy outcomes. Changes from baseline to 24 week in cognitive tests and neuropsychiatric rating scales. Data are n (%) and mean (SD).

	EGb 761^®^ (n = 97) [mean ± SD]	Standard treatment (n = 96) [mean ± SD]	*p*-value
MoCA total score	2.92 ± 3.90	1.33 ± 3.75	<0.005
MoCA delayed recall	0.88 ± 1.42	0.17 ± 1.39	<0.001
MoCA orientation	0.28 ± 0.75	−0.11 ± 0.75	<0.001
MoCA language	0.45 ± 1.05	0.09 ± 1.08	<0.05
HVLT total recall score	1.40 ± 5.47	−0.49 ± 5.20	<0.05
Shape trail test—trail 1	−38.2 ± 93.8	−15.6 ± 55.4	<0.05
Shape trail test—trail 2	−68.8 ± 174.5	−62.7 ± 174.9	n. s
Verbal fluency test	1.21 ± 6.14	−0.16 ± 4.29	n. s
WAIS-R DSST	7.22 ± 14.49	5.60 ± 14.36	n. s
HADS anxiety	−1.79 ± 3.72	−1.45 ± 4.05	n. s
HADS depression	−1.25 ± 4.74	−0.57 ± 4.17	n. s
NPI total score	−3.98 ± 13.86	−6.59 ± 55.68	n. s

**FIGURE 3:**
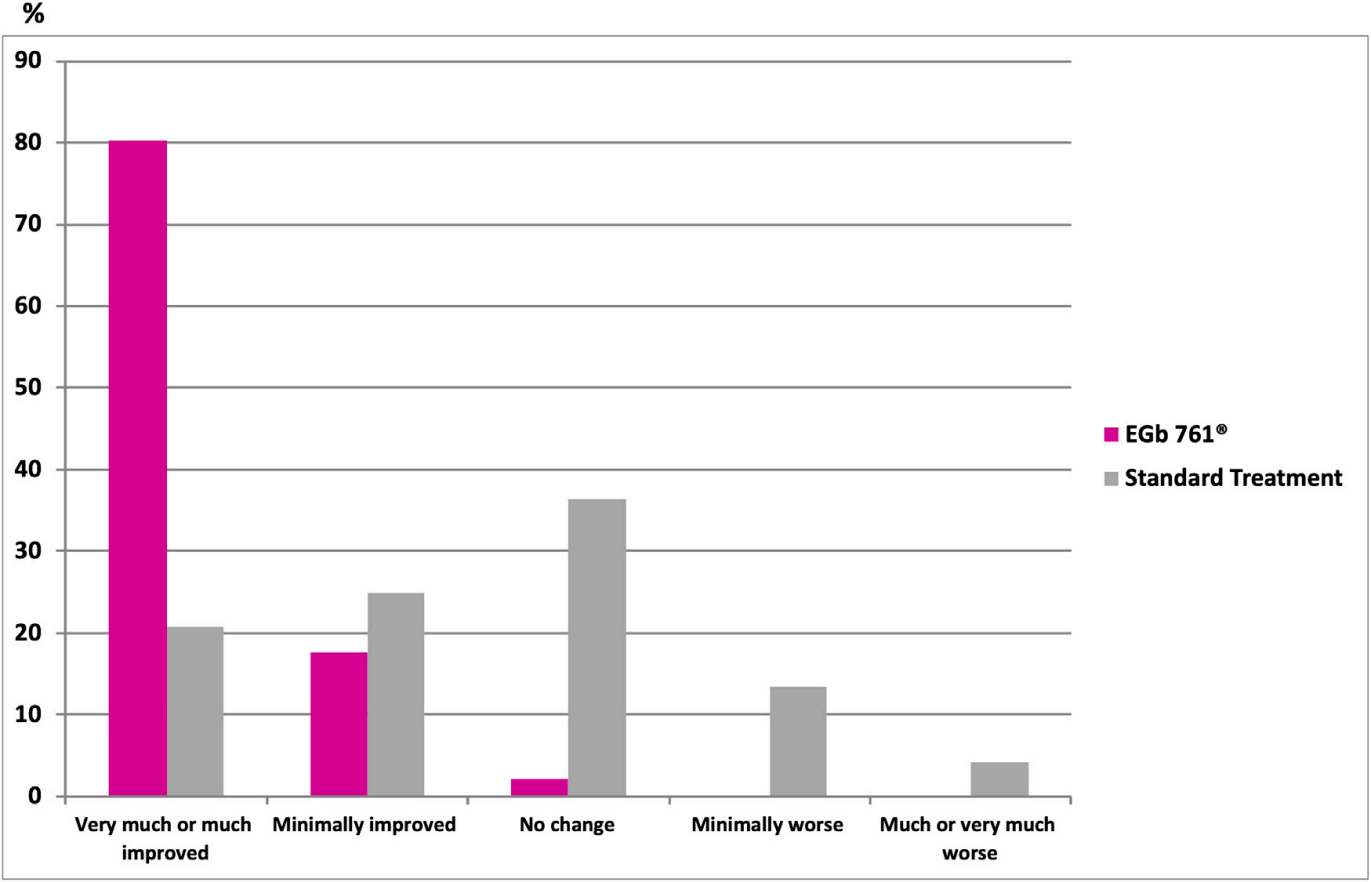
Clinical Global Impression of Change (CGI-C) at 24 weeks; *p* < 0.001 vs. standard (Fisher’s exact test two-sided).

Results involving the cognitive domains are presented in Table 2. The change score of the Hopkins Verbal Learning Test (HVLT) and the Shape Trail Test—Trail 1 at week 24 revealed that the drug-reference difference were 1.89 points (95% CI, 0.38 to 3.40; *p* < 0.05; Table 2) and −22.6 points (95% CI, −44.31 to −0.89; *p* < 0.05; Table 2). According to the clinicians’ global ratings (CGI-C), 80.2% of the patients treated with EGb 761^®^ improved much or very much compared to their baseline condition (Figure 3) versus only 20.8% of those who received standard treatment alone. The rate of recurrent strokes was low; only one recurrent stroke was observed in one patient of the EGb 761^®^ group, and three recurrent strokes were reported for two patients in the reference group. The analysis did not find significant difference for the Shape Trail Test Trail 2 (STT-2), the Verbal Fluency Test (VFT), the Digit Symbol Substitution Test (DSST) of the Wechsler Adult Intelligence Scale-Revised (WAIS-R), the Hospital Anxiety and Depression scale (HADS) and NPI in both groups at week 24.

### 3.3 Safety

Overall, 10.5% of patients experienced at least one adverse event during the 24-week randomized treatment period. The incidence of adverse events was similar between the two groups.16 adverse events were observed in 11 patients (11.1%) of the EGb 761^®^ group, and 14 adverse events were reported for 10 patients (9.9%) of the reference group. Most adverse events were mild and unlikely to be related to EGb 761^®^. The most frequently reported adverse event was nasopharyngitis, which was experienced by 3 patients (3%) in each treatment group. Other adverse events occurred in no more than 1% of patients in either group. Altogether, 5 serious adverse events were reported: 2 events (cerebral infarction, chronic kidney disease, one patient each) in the EGb 761^®^ group and 3 events (2 cerebral infarctions in one patient, lacunar infarction in one patient) in the reference group. A causal relationship could not be ruled out for three events: rash, dizziness (both possibly related) and chronic kidney disease (relationship unlikely). There were no clinically significant changes from baseline observed in vital signs, biochemical markers or electrocardiography results in either group.

## 4 Discussion

Considering the high incidence of stroke and the cognitive decline experienced by survivors in the Chinese population, there is a pressing need for novel interventions during the early post-stroke stage (Rajan et al., 2014); however, only few drugs have been dedicated to the topic. Our prospective randomized, multicenter, open-label trial focused on the effects of *Ginkgo biloba* extract EGb 761^®^ vis-à-vis cognitive function in patients after a recent mild-to-moderate ischemic stroke. The results provide valuable insight into the optimal selection of patients, efficacy measures and determining treatment duration for this disorder. Our study had an exploratory design. Evidence of previous trials has supported the use of EGb 761^®^ in the treatment of cognitive impairment (Gauthier and Schlaefke, 2014; Gavrilova et al., 2014). Thus we applied a cost-effective attempt to repurpose EGb 761^®^ at the same dose regimen to treat cognitive change after stroke. We adopted a 6-month treatment period because previous large-scale drug trials demonstrated that this interval was sufficient to observe symptomatic benefits in patients with vascular cognitive impairment (Ritter and Pillai, 2015).

We demonstrated that EGb 761^®^ exhibited slight improvements of cognitive performance, mainly documented by a larger increase in MoCA scores in the treatment group compared with the reference group. MoCA has been found sensitive when it comes to identifying changes in cognitive function, especially in executive deficits that add to the outcome prediction of post-stroke cognitive impairment and post-stroke dementia (Shopin et al., 2013; Dong et al., 2014; Burton and Tyson, 2015; Ritter and Pillai, 2015; Tan et al., 2017). In our study, we only enrolled patients with mild neurological deficits because they had to be able to participate in functional cognition evaluation: a ceiling effect can therefore not be ruled out while explaining the similar high response in both groups after 24 weeks. Nevertheless, the difference between drug and reference treatment, although minor, stayed in line with the typical results of other vascular cognitive impairment trials (Guekht et al., 2017; Nijsse et al., 2017; Chabriat et al., 2020), providing further evidence that the MoCA is a useful instrument to assess cognitive outcomes after mild stroke. It is noteworthy that both our trial and the findings from the previous study (Li et al., 2017) have shown that Ginkgo biloba extract as an add-on to the standard treatment promoted MoCA improvement at 6 months. Comparatively, our current research used lower EGb 761^®^ dosages (240 mg daily) compared to Li et al. (450 mg daily). While several studies have indicated that EGb 761^®^ may increase the risk of bleeding by inhibiting platelet aggregation and platelet activating factor function (Kudolo et al., 2002; Bent et al., 2005), our results supported that the use of lower dosage of EGb 761^®^ (240 mg/d) did show clinical benefits in cognitive performance within a 24-week period and alleviated the concern of bleeding in future study designs. A long-term study lasting for 1–3 years would help to fully explore the efficacy of EGb 761^®^ in preventing post-stroke dementia. Strengths of our study also included the exclusion of patients who take antidementia drugs, psychoactive drugs and so forth regularly to avoid possible confounding in the result of the trial on the cognitive function.

In particular, the cognitive function of patients in the reference group did improve to some extent at 24 weeks, confirming the benefits of secondary prevention, including risk-factor management. It has been postulated that risk-factor management could restore cognitive performance and NPS. Therefore, we recommend that future analyses also include medical history, concomitant disease, as well as significant laboratory values such as blood pressure, blood glucose, and blood lipid during treatment. A trend towards a progress in learning and memory along with executive functions was also detected with the EGb 761^®^ group versus control. Furthermore, there is a marked and statistically significant difference in favor of EGb 761^®^ treatment in terms of the clinicians’ global judgment of change on the patients’ overall condition. This may be somewhat overestimated: if there is any doubt in an open-label study, it is entirely possible that the better of two possible ratings was chosen if a patient had more intensive treatments. Addressing additional aspects of quality of life or instrumental activities in further studies may provide more insight. Other secondary outcomes assessed did not achieve clinical significance and our analysis demonstrated that EGb 761^®^ did not improve psychological outcomes by concomitant treatment in our pilot period. The threefold higher number of recurrent strokes in the standard treatment group might be a hint towards a preventive effect of EGb 761^®^ treatment. However, due to the very low rate of recurrence, this data point has to be interpreted with caution. In terms of safety, the application of EGb 761^®^ was well tolerated, which was in line with an established safety profile in the treatment of dementia (Gauthier and Schlaefke, 2014; Gavrilova et al., 2014) as well as a low incidence of adverse events in this study population.

The findings above were not representative of patients with more severe stroke sequelae due to our restrictive inclusion criteria. Indeed, patients tend to develop focal neurologic symptoms like aphasia or paralysis after a stroke. Despite a higher risk of post-stroke cognitive deterioration, these patients are not likely to be enrolled due to several reasons: they may not able to understand and complete the cognitive assessments; they could also be hospitalized in a long-term care, which may not allow sufficient time for them to be identified and enrolled; they are also likely to comorbid other serious medical conditions that are inappropriate for clinical trials. These factors limit the generalizability of study population, making it challenging to gain valid evidence towards the effects of EGb 761^®^ on patients with severe cognitive impairment. As expected, most patients enrolled in our groups had only mild neurological deficits as well as mild cognitive decline. Considering the slow nature of cognition-associated decline after stroke, the cognitive state of those patients might stay normal or stable during a 24-week follow up (an interval which might only represent the early warning stage of post-stroke cognitive impairment). In general, pharmacological intervention at the early stage of associated diseases is more effective in preserving cognitive function than delayed treatment (Lissek and Suchan, 2021; Marcolini et al., 2022). Ginkgo biloba extract contains several compounds that have been shown to improve blood flow to the brain, reduce oxidative stress and enhance the activity of neurotransmitters such as acetylcholine, which is important for learning and memory (Ahlemeyer and Krieglstein, 2003). Therefore, the neuroprotective effects of EGb 761^®^ could produce a modest yet consistent benefit in slowing or halting the progression of cognitive decline, rather than reversing it once it has already developed into dementia.

Moreover, it is noteworthy that most adopted cognitive tests in current vascular dementia trials have much in common with the assessment tools in patients with Alzheimer’s disease (AD). These traditional screening measures seem general but presumably lack sensitivity in detecting the early subtle changes in cognition after stroke. Indeed, those insufficient evaluations of AD-related abnormalities might also confound our results, since vascular and degenerative factors always interact and exacerbate cognitive deterioration together in the long term (Korczyn, 2002). Thus more attention is needed to develop more specific cognitive tests for assessments after stroke, taking into account the fact that cognitive injuries vary, depending on different ischemic types including stroke location, volume, number of incidents and severity (Kalaria et al., 2016).

Additional limitations of our study include the open-label design and the lack of placebo, which has the potential of bias and would have made the study not feasible, especially in clinical event reporting and ascertainment. However, given the highly objective nature of cognitive tests, it is unlikely that the performance of our outcome measures of main interest were adversely affected. Another limitation might be the absence of biomarker evaluation related to cognitive status, which could help the assessment of cognitive function. In addition, we acknowledge the lack of diet regulation among the participants and this variability could have influenced the post-stroke recovery as well as the pharmacokinetics of the drug, potentially impacting the study outcomes. Moreover, the duration of our study—only 24 weeks—may have been too short to reveal the potential of EGb 761^®^ in attenuating the decline in cognitive function, since cognitive deficits in patients after stroke tend to develop slowly. Larger samples and longer follow-up for 1–3 years might be required in future studies for major post-stroke cognitive impairment to manifest. In conclusion, evidence from our trial suggests that EGb 761^®^ 240 mg/d showed clinical benefits in cognitive functioning of patients compared with standard care after a mild-to-moderate ischemic stroke. Future randomized controlled trials with a larger sample size and higher statistical power may help further establish the inclusion criteria and treatment duration; they can also further validate the effects of EGb 761^®^ in patients with a broader range of severity of cognitive impairment.

## Data Availability

The original contributions presented in the study are included in the article/Supplementary Material, further inquiries can be directed to the corresponding author.
