# Supplementary material for: Ginkgo biloba extract EGb 761® improves cognition and overall condition after ischemic stroke: Results from a pilot randomized trial
Source: Front Pharmacol. 2023 Mar 29;14:1147860. doi: 10.3389/fphar.2023.1147860 (PMC10090660; doi:10.3389/fphar.2023.1147860)
Supplement: Supplementary file 1 [file Table1.DOCX]

Supplementary Material

*Ginkgo biloba* extract EGb 761^®^ improves cognition and overall condition after ischemic stroke: Results from a pilot randomized trial

Mei Cui, Tongyao You, Yuwu Zhao, Ruozhuo Liu, Yangtai Guan, Jianren Liu, Xueyuan Liu, Xin Wang, Qiang Dong*

*** Correspondence:** Qiang Dong: dong_qiang@fudan.edu.cn

# Supplementary Tables

**Table S1.** Full list of investigators and study centers.

| **Investigator** | **Center number, address** |
| --- | --- |
| Dr. Dong Qiang | Department of Neurology, Huashan Hospital, Fudan University, No.12 Middle Wulumuqi Road, Shanghai, 200040, China; |
| Dr. Guan Yangtai | Department of Neurology, ChangHai Hospital, Naval Medical University, No.168 Changhai Road, Shanghai, 200433, China |
| Dr. Liu Xueyuan | Department of Neurology, Shanghai Tong Ji University Affiliated Tenth People’s Hospital, No.301 Yanchang Middle Road, Shanghai, 200072, China |
| Dr. Wang Xin | Department of Neurology, Zhongshan Hospital, Fudan University, No.180 Fenglin Road, Shanghai, 200032, China |
| Dr. Zhao Yuwu | Department of Neurology, Shanghai Jiao Tong University Affiliated Sixth People’s Hospital, No.600 Yishan Road, Shanghai, 200233, China |
| Dr. Liu Jianren | Department of Neurology, Shanghai Jiao Tong University Affiliated Ninth People’s Hospital, No.639 Zhizaoju Road, Shanghai, 200011, China |
| Dr. Liu Ruozhuo | Department of Neurology, Chinese People’s Liberation Army General Hospital, No.28 Fuxing Road, Beijing, 100039, China |
